# Anti-*Candida albicans* IgG Antibodies in Children With Autism Spectrum Disorders

**DOI:** 10.3389/fpsyt.2018.00627

**Published:** 2018-11-26

**Authors:** Heather K. Hughes, Paul Ashwood

**Affiliations:** Department of Medical Microbiology and Immunology, and The Medical Investigation of Neurodevelopmental Disorders (M.I.N.D.) Institute, University of California, Davis, Davis, CA, United States

**Keywords:** autism, Candida, antibodies, immunity, behavior

## Abstract

The gut microbiota are known to have a profound influence on both mucosal and systemic immunity and are important for gastrointestinal (GI) function. In addition, new evidence shows that the microbiota significantly influence neurodevelopment and behavior. Immune dysfunction and GI distress are extremely common in individuals with autism spectrum disorders (ASD). A growing body of evidence suggests that individuals with ASD have significant aberrations in the composition of their gut microbiota, known as dysbiosis. However, these studies have focused on the bacterial components of the microbiota, leaving the fungal microbiota in ASD poorly studied. Increases in fungal species such as *Candida albicans* are associated with inflammatory bowel disorders, and have recently been implicated in several neurological disorders including schizophrenia. We aimed to determine if children with ASD exhibit elevations in antibodies that target *C. albicans*, indicating current or previous overgrowth of this fungal species. We measured anti-*C. albicans* immunoglobulin (IgG) in plasma from 80 children enrolled in the UC Davis MIND Institute CHARGE study. Measurements were acquired using a commercial ELISA kit. Plasma anti-*C. albicans* antibody positivity was found in 36.5% (19/52) of children with ASD. Anti-*C. albicans* antibodies in typically developing controls was (14.3%; 4/28). Overall, ASD children had a higher rate of high-positive values compared to typically developed children with an unadjusted odds ratio of 3.45 (95% confidence interval, 1.0409 to 11.4650; *p* = 0.041, two-tailed). GI dysfunction was found in about half of the ASD children who were positive for anti-*Candida* IgG. This study provides evidence of a new microbial risk factor for ASD.

## Introduction

Autism spectrum disorders (ASD) are a group of heterogeneous neurodevelopmental disorders defined by deficiencies in social interactions, cognition and communication, and stereotypical and repetitive behaviors (1). Although specific genetic mutations account for 10–20% of causes for ASD, heritability risks are high and could include genes that lead to susceptibility for environmental exposures, or similar epigenetic mechanisms brought about by shared household exposures to environment (2). Environmental risk factors for having a child with ASD include gestational exposure to pollution and pesticides, and maternal infections and inflammation during pregnancy (3–5). ASD is also linked to familial autoimmunity and asthma (6–8) and many individuals with ASD have significant immune dysfunction (9, 10). Gastrointestinal (GI) dysfunction is also common in these individuals, with at least 50% of individuals with ASD experiencing GI issues (11).

Gastrointestinal and immune dysfunction has been linked to aberrant composition of the microbiota, known as dysbiosis (12). Recently, researchers have identified significant bacterial dysbiosis in individuals with ASD (13–23). Bacterial dysbiosis can be caused by a variety of insults including frequent antibiotic use, which can also increase the risk of fungal overgrowth in the GI tract. *Candida albicans* is generally considered a passive commensal yeast of the GI and genitourinary tracts, however, it has polymorphic capabilities and under certain conditions, including altered competition within the gut, it is capable of transitioning to its pathogenic and invasive fungal form (24). The presence of *Candida* species during recolonization after antibiotics also contribute to dysbiosis (25) and are associated with GI disorders such as celiac disease and inflammatory bowel disorders (26–28). These competitive relationships of bacterial versus fungal microbiota are complex and still being investigated, as newer techniques develop to identify them (24).

Overgrowth of *Candida* species has been noted in ASD in a few studies utilizing culture-based techniques (29–31) and more recently in a study using sequencing techniques, which found *Candida* to be present in the stool of ASD children in nearly twice the numbers of typically developed children (21). Elevations of d-arabinitol, a suspected metabolic byproduct of *Candida* species, was found in a study of 21 Italian children with ASD (32). D-arabinitol was also significantly reduced in ASD children after probiotic administration, and this correlated with improved behaviors including ability to concentrate (33). Fungal infections are an emerging area of research interest in ASD, and exposure can also be identified by looking at immunoglobulin (Ig) that target fungal antigens. They may be present in individuals with dysbiosis, as this may lead to breeches in intestinal barrier function and subsequent immune responses to commensal microbiota, including the production of IgG antibodies indicating current or previous overgrowth of this fungal species (34, 35). In schizophrenia, significantly elevated IgG antibodies to fungal microbiota have been seen, especially in males, however, they were also seen in bipolar females associated with lower cognitive scores (36). So far these antibodies have not been studied in individuals with ASD, therefore we aimed to determine if similar antibodies are over-represented in ASD children with and without GI dysfunction compared to their typically developing (TD) counterparts.

## Methods

### Study participants

Eighty participants ranging in age from 3 to 13 years old were enrolled in this study as part of the larger population based cohort Childhood Autism Risk from Genetics and Environment (CHARGE) study (37). ASD diagnoses were confirmed using the Autism Diagnostic Interview-Revised (ADI-R), and the Autism Diagnostic Observation Schedule (ADOS) at the time of enrollment. Social Communication Questionnaire (SCQ) was used to screen for characteristics of ASD in the typically developed children. Criteria for enrollment in the typically developed groups were scores of below 15 on the SCQ and above 70 on the Mullen Scales of Early Learning (MSEL) and Vineland Adaptive Behavior Score (VABS). There were *n* = 52 ASD subjects (median age 7.42 years (IQR: 5.17–9.42); 8 females) and *n* = 28 TD (median age 6.5 years (IQR: 5.58–8.33); 3 females). All subjects were administered the Aberrant Behavior Checklist (ABC) assessment. Parents also completed a CHARGE GI history (GIH) survey and GI symptom survey, based upon Rome III Diagnostic Questionnaire for the Pediatric Functional GI Disorders (22, 38) to identify symptoms of GI dysfunction including abdominal pain, gas/bloating, diarrhea, constipation, pain on stooling, vomiting, sensitivity to foods, difficulty swallowing, and blood in stool or vomit. Participants were excluded if they had inflammatory bowel disease (IBD) or other GI pathology, recent evidence of a GI infection and/or were taking medication that might alter GI function such as stool softeners which can alter motility or recent antibiotics/antifungals that can induce dysbiosis. In addition, participants with seizure disorder, genetic disorders, or other chronic diseases and/or infections were also excluded.

This study was approved by institutional review boards for the State of California and the University of California, Davis. Both written and informed consent was obtained from a legal guardian for all study participants prior to data collection in accordance with the UC Davis IRB protocol.

### Blood collection and enzyme-linked immunosorbent assay

Peripheral blood was collected from each subject in acid-citrate dextrose Vacutainers (BD Biosciences; San Jose, Ca). Blood was centrifuged and plasma harvested and stored at −80°C until time of assay. Human anti-*Candida albicans* IgG was measured using enzyme-linked immunosorbent assay with a commercially available kit (Abcam, Cambridge, MA, USA) following the manufacturer's instructions. According to manufacturer's recommendations, 96 well plates were pre-coated with *Candida* capture antigens. Plasma samples were diluted 1:100 with diluent provide, and 100 μL of diluted plasma, positive, negative, and cut-off controls were added to the plate in duplicate, leaving two blank wells per plate, and incubated in the dark for 1 h at 37°C. Wells were aspirated and washed three times with provided washing solution. 100 μL *Candida albicans* anti-IgG HRP Conjugate was added to all wells except for blank wells, and incubated for 30 min in the dark at room temperature, then washed again three times. One hundred microliter 3,3',5,5'-tetramethylbenzidine (TMB) solution was added to the wells and incubated for 15 min, the reaction was stopped with reagent provided by the manufacturer. Absorbance was measured immediately on spectrophotometer at 450 nm with dual wavelength, using 620 nm as reference wavelength.

### Statistics

Positivity was identified using background-adjusted absorbance of provided cut-off control. Background-adjusted sample absorbance above the positive control are denoted as high-positive. Comparison of qualitative variables between groups was assessed using Fisher's Exact Probability test with probability (P) of < 0.05 considered significant.

## Results

**Figure 1:**
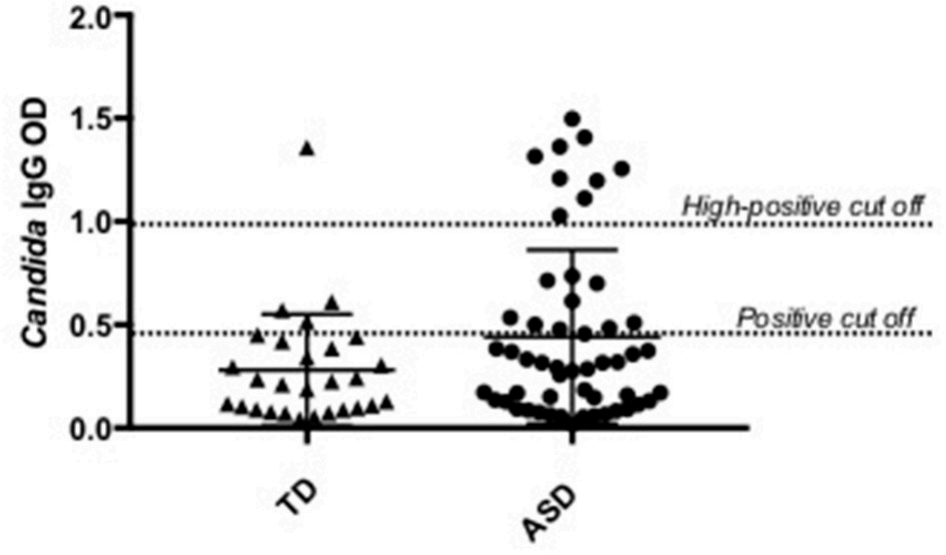
*Candida albicans* plasma positivity in ASD vs. typically developing children. Nineteen children with ASD were positive for *C. albicans* IgG antibodies compared to four typically developing children.

Plasma anti-*C. albicans* antibody positivity was found in 36.5% (19/52) of children with ASD. There was a significantly higher percent positivity of plasma anti-*C. albicans* antibodies in children with ASD than in healthy TD controls (14.3%; 4/28), with an unadjusted odds ratio of 3.45 (95% confidence interval, 1.040 to 11.465; *p* = 0.041, two-tailed; Table 1). Nine of the 19 positive samples in children with ASD were considered high-positive, versus only one high-positive in the TD population (Figure 1). When examining ASD children with positivity for anti-*Candida* IgG, 9 of 19 (47%) had symptoms of GI dysfunction, of the 33 negative for anti-*Candida* IgG 13 had GI symptoms (39%), however no significant differences were observed based on presence or absence of GI symptoms in children with ASD. In comparison, 1 of 4 TD children positive for anti-*Candida* IgG had GI issues (25%) and only 5 of 24 who were negative for anti-*Candida* IgG (21%), however, these data are limited by small sample size of the TD group with GI symptoms.

**Table 1:** The frequency of plasma anti-*Candida albicans* antibodies in children with ASD compared to typically developed children.

**Study group**	**Anti-*C albicans***	**Anti-*C albicans***
	**IgG positive**	**IgG negative**
ASD	19	33
	36.5%	63.4%
TD	4	24
	14.3%	85.7%
***P value***	**0.04**
**OR (95% CI)**	**3.45**

*OR, odds ratio*.

## Discussion

Study of the microbiota-gut-brain axis has exploded over the past few decades, however, fungal microbiota research is still in the early stages. Fungal overgrowth has been implicated in inflammatory bowel disorders (39–41), schizophrenia and bipolar disorder (36), and was recently discovered within the cells of post-mortem brain tissue in Alzheimer's patients (42). Our results indicate that children with ASD have elevations in IgG against this fungal commensal, likely indicating an overgrowth of *Candida albicans* within the GI tract. The source of dysbiosis and *Candida* overgrowth in ASD is currently unknown but may be influenced by the immune dysfunction seen in many children with ASD. Alternatively, this overgrowth may be contributing to immune and GI dysfunction and the behaviors seen in ASD. This raises questions as to whether dysbiosis is present very early in life in ASD and may be involved in the etiology of ASD that need further investigation. IgG antibodies to commensal organisms become more common as individuals age, possibly due to transient intestinal breeches that might occur during enteric infections or dysbiosis from antibiotics, therefore identifying earliest exposure is a key next step to this research. Future research should attempt to identify presence of IgM antibodies which indicate current or very recent infection (35), ideally during infancy and/or early life to see if there is a relationship between infection and ASD symptom emergence.

The presence of *Candida* species is known to alter the assembly of microbiota after antibiotics, contributing to dysbiosis in mice (25). This suggests that if present, a different bacterial composition will occur than when absent. Therefore, if *Candida* is present in very early life it could interfere with initial colonization and the successions of composition during the early transitional stages of microbiota development leading to dysbiosis (43). The early microbiota are typically inherited from maternal sources, especially during vaginal delivery (43). If *Candida* is overgrown in mother during gestation, it could potentially be passed on to offspring as an early colonizer, interfering with normal colonization. Furthermore, the immune response to fungal overgrowth includes elevations of interleukin (IL)-17 (44). This cytokine is implicated in maternal immune activation (MIA) models of ASD, and without it ASD behaviors in MIA offspring were absent (45, 46). Further research utilizing animal models of fungal dysbiosis in early life or gestation could help determine the degree in which fungal microbiota may be contributing to the dysbiosis and behavioral abnormalities in offspring.

To our knowledge, this is the first study to look at anti-*Candida albicans* IgG in children with ASD. Our recent work identified an imbalance in inflammatory versus regulatory cytokines such as transforming growth factor (TGF) beta, with alterations in the microbiota in this same population based cohort (22). Although GI symptoms were not strongly associated with *Candida* IgG positivity, we previously found microbiota changes in individuals with ASD irrespective of GI symptoms (22). This suggests that dysbiosis could occur even in the absence of GI symptoms. This preliminary study was initiated to guide future research on the fungal microbiota and ASD. Further validation of our results could include exploring fungal composition within the gut as well as metabolic byproducts of yeast species such as d-arabinitol and ethanol, and identifying associations these might have with behaviors in ASD.

## Ethics statement

This study was carried out in accordance with the recommendations of University of California Davis, IRB committee with both written informed consent from all subjects. All subjects gave written informed consent in accordance with the Declaration of Helsinki. The protocol was approved by the IRB committee at UC Davis.

## Author contributions

All authors listed have made a substantial, direct and intellectual contribution to the work, and approved it for publication.

### Conflict of interest statement

The authors declare that the research was conducted in the absence of any commercial or financial relationships that could be construed as a potential conflict of interest.
